# Longitudinal detection of prion shedding in nasal secretions of CWD-infected white-tailed deer

**DOI:** 10.1099/jgv.0.001825

**Published:** 2023-01-27

**Authors:** Caitlyn N. Kraft, Nathaniel D. Denkers, Candace K. Mathiason, Edward A. Hoover

**Affiliations:** ^1^​ Prion Research Center, College of Veterinary Medicine and Biomedical Sciences, Department of Microbiology, Immunology, and Pathology, Colorado State University, Fort Collins, CO 80523, USA

**Keywords:** CWD, nasal swabs, longitudinal secretion, IOME, RT-QuIC

## Abstract

Chronic wasting disease (CWD) is an emergent prion disease spreading in cervid populations in North America, South Korea and Scandinavia. Rapid detection of CWD prions shed by live animals using minimally invasive methods remains an important need. Previous studies in deer, elk and hamsters have demonstrated prion replication in the nasal olfactory mucosa, yet the temporal profile of CWD prion shedding in nasal secretions has not been well characterized. Here we report nasal prion shedding in 18 deer orally exposed to low doses of CWD prions and monitored longitudinally by several parameters. Serially collected nasal swabs were assayed for CWD prion seeding activity using iron oxide magnetic extraction and real-time quaking-induced conversion (IOME RT-QuIC). These findings were correlated with the results from longitudinal tonsil biopsies, terminal tissues and PRNP genotype. We detected nasal prion shedding 3–16 months after the first positive tonsil biopsy in ten of the 18 deer; detectable shedding persisted thereafter in nine of the ten animals. Surprisingly, nasal swabs were negative in eight deer, even though all were CWD-infected as determined by tonsil biopsies and terminal tissue assays. Nasal shedding was detected more often in deer that were homozygous for glycine at codon 96, and those that were near or demonstrating symptoms of clinical disease shed earlier and more frequently, irrespective of prion exposure dose. The results of this study demonstrate nasal shedding of CWD prions that can be detected using minimally invasive nasal swab sampling and RT-QuIC analysis.

## Introduction

Chronic wasting disease (CWD) is an emerging transmissible spongiform encephalopathy (TSE) that affects cervids (moose, elk and deer) [1], and the only TSE known in free-ranging animals. Available information infers that CWD is transmitted by direct or indirect contact with prions shed in secreta and excreta (i.e. urine, faeces, saliva, blood) [2–4]. Ante-mortem diagnosis of CWD requires biopsy of tonsil or recto-anal lymphoid tissue, or post-mortem collection of lymph node or brain for testing by immunohistochemistry (IHC) or ELISA. Methods for rapid detection of clinically silent CWD in living animals using less invasive methods remain an important need in CWD surveillance and management.

Studies of transmissible mink encephalopathy in hamsters have demonstrated prion infectivity in nasal lavage fluid and that prion disease can be transmitted by extra-nasal droplet inoculation [5]. CWD prion seeding activity has been demonstrated in the posterior nasal fossa by deep nasal brush sampling in elk and deer [6, 7]. The complex nasal turbinate anatomy in cervids also makes sample collection using long cervical brush swabs complicated. Thus, more distal sampling with more simple nasal swabs would offer a more tractable and practical clinical sampling.

Methods to detect prion seeding activity in complex biofluids using real-time quaking-induced conversion (RT-QuIC) have progressed, and have been advanced by use of iron-oxide bead magnetic extraction (IOME) [6–13]. In the current study we sought to gain insight into CWD transmission through a longitudinal profile of nasal prion shedding using a simplified sampling procedure, and by correlating these results with evidence of CWD in tissues of the same animals.

## Methods

### Study design

A total of 20 hand-raised, CWD-free white-tailed deer were sourced through collaborations with the Warnell School of Forestry and Natural Resources, University of Georgia, and housed in the indoor CWD research facility at Colorado State University. Cohorts of deer were exposed by oral [*per os* (PO)] instillation of inoculum. Deer were inoculated with aliquots of CWD-positive brain homogenate pool or saliva pool, each of which contained previously determined amounts of prion seeding activity (as determined by RT-QuIC and expressed as milli- or micrograms of CWD-positive brain tissue) [14, 15] (Table 1).

**Table 1.** Comparison of positivity of brain obex (S8), brain frontal lobe (S1), olfactory bulb and terminal nasal swab samples by RT-QuIC Positive samples highlighted in green are denoted by the number of replicates positive by RT-QuIC. Samples were considered positive using an unpaired *t* test to negative plate controls. *P<0.05, **P< 0.01, ***P< 0.001. Genotype for codon 96, months post inoculation (MPI) and clinical status are noted for each deer. N/A, missing sample.

	Clinical status		Brain [obex; S8]	Brain [frontal; S1]	Brain [olfactory]	Terminal nasal swabs
ID no. (96)	MPI	+/−	Pos/total	Pos/total	Pos/total	Pos/total
**CWD(+)**						
1308 (GG)	18	+	8/8 ***	8/8 ***	8/8 **	8/8 ***
1303 (GG)	22	+	8/8 ***	8/8 ***	8/8 ***	6/8 **
1031 (GG)	32	+	8/8 ***	8/8 ***	8/8 **	8/8 ***
1081 (GG)	20	+	8/8 ***	8/8 ***	n/a	8/8 ***
1309 (GG)	28	–	8/8 ***	8/8 ***	8/8 *	8/16 *
1082 (GG)	24	+	8/8 ***	8/8 ***	8/8 ***	8/8 ***
1311 (GG)	37	+	8/8 **	8/8 ***	8/8 ***	7/8 ***
1093 (GG)	23	+	8/8 ***	8/8 ***	8/8 ***	6/8 **
1076 (GG)	22	+	7/8 **	8/8 ***	8/8 ***	8/8 ***
1078 (GG)	24.5	+	8/8 ***	8/8 ***	8/8 **	8/8 ***
1079 (GG)	32	+	8/8 ***	8/8 ***	8/8 **	2/8
1313 (GG)	25	+	8/8 ***	8/8 ***	8/8 ***	2/8
1306 (GS)	47	+	8/8 ***	7/8 ***	8/8 ***	4/12
1315 (GS)	39	+	8/8 ***	0/8	0/8	0/8
1316 (GG)	23	–	8/8 **	3/8	0/8	0/8
1310 (GS)	28	–	8/8 ***	2/8	1/8	0/8
1305 (GS)	28	–	6/12 *	0/8	1/8	0/8
1307 (GS)	28	–	2/12	0/8	5/8	n/a
**CWD(-)**						
1437 (GG)	24	–	0/8	0/8	0/8	0/8
1444 (GG)	24	–	4/8	1/8	0/8	0/8

One cohort of deer (*n*=2) from the same source (Table 1) served as the negative controls that were inoculated with brain homogenate or saliva from known CWD-negative deer [15]. The PRNP codon 96 genotype of each deer is reported (Table 1), as this polymorphism is known to affect the rate of infection progression (*n*=15 deer were 96GG; five were 96GS). The time each deer was on the study is represented by months post-inoculation (MPI) in Table 1. Clinical disease status was monitored and recorded using a scoring system throughout the duration of the study [13].

### Sample collection and processing

**Fig. 1.**
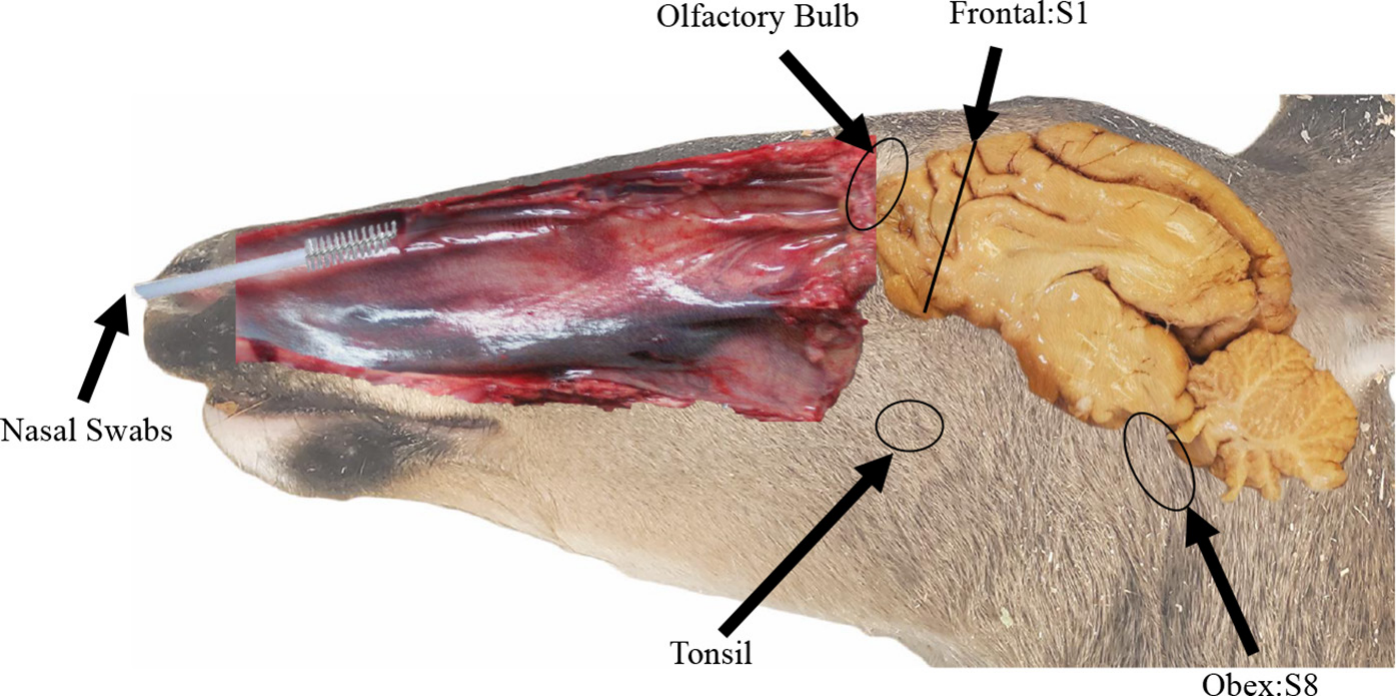
White-tail deer brain sectioning for post-mortem processing. The caudal aspect of the frontal lobe (section 1) and obex (section 8) were analysed by RT-QuIC. Prion positivity has been shown to progress from caudal to rostral across the brain. Progression of CWD was characterized from tonsil, to S1 brain section/olfactory bulb, and to nasal swab secretions collected by cytology brush.

We sampled nasal secretions at 2- or 3-month intervals from the time of inoculation to time of necropsy (3–48 months). Secretions were collected using sterile 8-inch cytology brushes (Fisher Scientific) inserted ~2 inches into the nasal vestibule (Fig. 1), then spun to collect fluids and nasal superficial mucosal cells.

Swabs that were contaminated with blood were excluded from the study. Two brushes per nostril were collected and all four were combined into 1 ml PBS. Sample tubes were vortexed, swabs removed and cell/fluid suspensions stored at −80 °C until assayed.

All deer were monitored for evidence of CWD infection using contemporary methods. Tonsil biopsies were collected every 2 or 3 MPI and assayed for prion seeding activity by RT-QuIC (see below) and for PrP^CWD^ deposition by IHC as previously described [16]. At necropsy, a standard array of multiple tissues were collected and analysed as above. To assess the presence of neuro-invasion, sections of the obex region of the medulla [section 8 (S8)], frontal cortex [section 1 (S1)] and olfactory bulbs were collected at time of necropsy and analysed by RT-QuIC. Samples of all tissues analysed by RT-QuIC were homogenized in 1× PBS at a 10 % (w/v) ratio, using a Bead Ruptor Tissue Homogenizer (Omni International), then stored at −80 until assayed. Terminal tissues were fixed in periodate-lysine-paraformaldehyde (PLP), processed and analysed by IHC as previously described [16].

### RT-QuIC analysis

Given that levels of CWD prion seeding activity in excreta are several logs lower than those in brain or lymphoid tissues, all nasal samples were assayed using the IOME RT-QuIC protocol [10]. Nasal swab samples were diluted 1 : 100 in PBS to a total volume of 0.5 ml and extracted using IOME. Two microlitres of iron oxide beads (Bangs Laboratories) were added to each sample then rotated end-over-end for 30 min, after which the beads were magnetically separated, then resuspended in 10 µl 1× PBS containing 0.1 % SDS. Olfactory bulb and brain section homogenates were diluted to a 10^−5^ final dilution in 1× PBS containing 0.1 % SDS. Samples were distributed in quadruplicate in 96-well plates (Greiner Bio-One; VWR) loading 2 µl per well, into which 98 µl of RT-QuIC reaction mixture containing recombinant Syrian hamster prion protein (rhaPrP: amino acids 90–231) [9, 17] was added. Replicates were considered positive when relative fluorescence units exceeded five standard deviations above the baseline mean fluorescence [18]. A minimum of eight replicates were assayed for each sample. Samples were statistically analysed using GraphPad Prism software, and compared to negative plate controls using the Mann Whitney unpaired *t*-test. Samples with a *P* value <0.05 were considered positive.

## Results

**Fig. 2.**
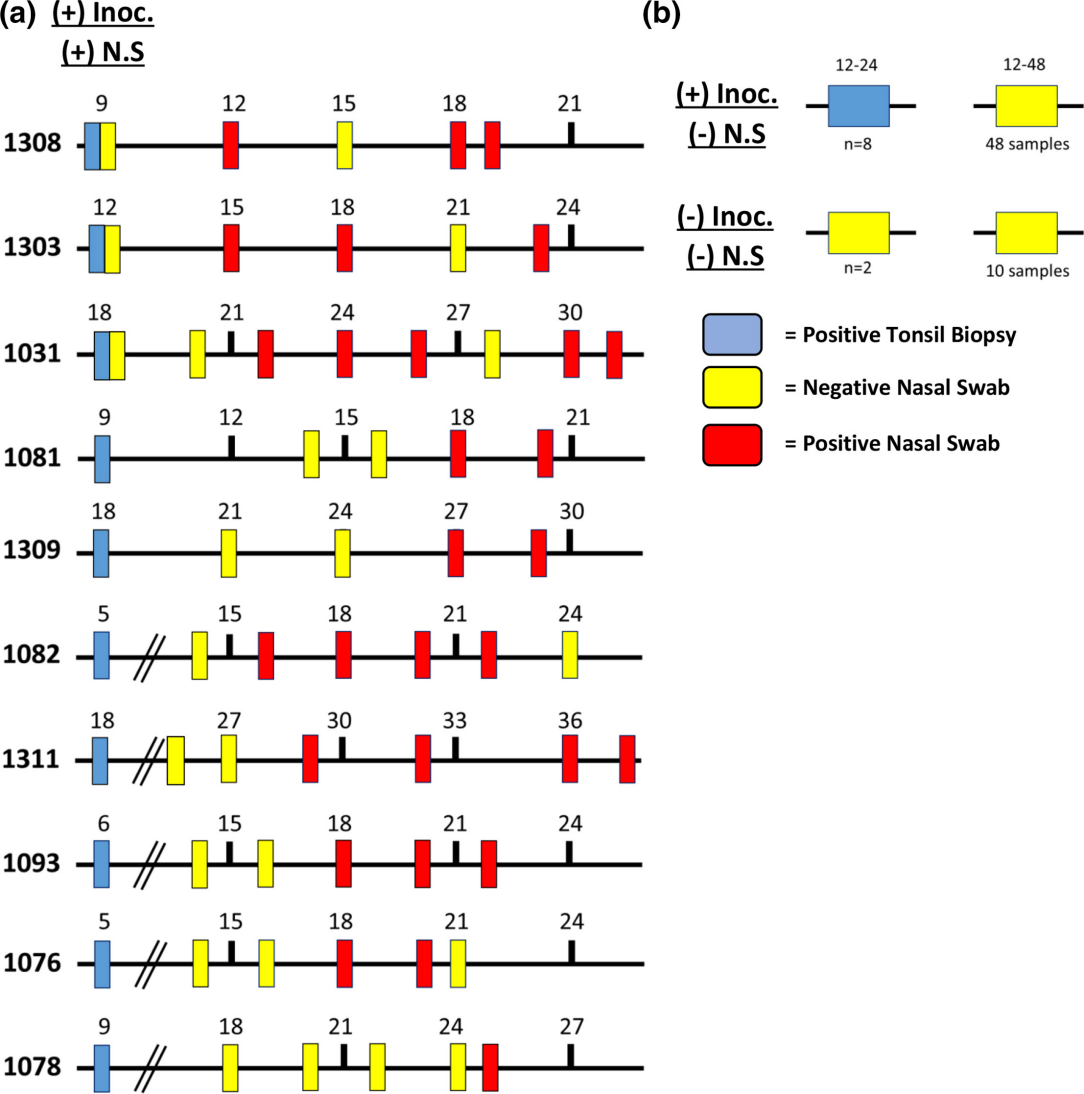
(a) Longitudinal timelines of subsections of 10 deer inoculated with 10 mg, 1 mg or 300 ng brain or saliva material. Terminal and longitudinal nasal swab samples were analysed by RT-QuIC (positive nasal swab=red tick mark, negative nasal swab=yellow tick mark) and compared to the first IHC(+) tonsil biopsy (blue tick mark). (**b**) Positively inoculated deer (*n*=8) with IHC(+) tonsil biopsy (range: 12–24 MPI) and negative in all nasal swab samples (*n*=48). Negative controls (*n*=2) remained negative in all samples (*n*=10).

To determine the longitudinal shedding profile in white-tailed deer nasal secretions, we assayed nasal swabs from each animal in reverse chronological order, beginning with the terminal collection point and assayed until two consecutive swabs from a given animal were consistently negative. We correlated those results with those from serially collected tonsil biopsies (Fig. 2).

We have previously shown that lower CWD challenge doses prolong the detectable onset of infection [15]. Positive tonsil biopsies were recorded in 18 of 18 (100 %) CWD-inoculated deer, with the time of first positive assay varying from 5 to 24 months post-exposure. Nasal shedding of prion seeding activity was detected only after CWD seeding activity was established in the tonsil. Positive nasal swabs were detected in 10 of the 18 deer (Fig. 2). Nasal shedding was detected as early as 3 months (range: 3–16 months; mean=9.1) after the first PrP^CWD^ deposit was recognized in the tonsil. In four of the ten deer, once shedding was detected, it persisted for the duration of the study. A discontinuous pattern of positivity was observed in five deer, and one deer was positive only at terminal collection. Detectable shedding continued for an average of 5.22 months (range: 2–10 months) after first positive activity. Terminal nasal swab collections were positive in eight of the ten deer that had previous positive samples but was not detected in the other eight deer. Seeding activity was not detected in negative control deer (*n*=2) in any of the nasal secretions or tonsil biopsies assayed by RT-QuIC and IHC.

To gain insight into the progression and potential origin of CWD prions in the nasal cavity, we compared nasal swab positivity with that of three regions of brain (S8, S1 and olfactory bulb). In all cases in which prion seeding activity was detected in nasal secretions, regardless of the time-point, tonsil and all brain tissues were also positive (Table 1). There were three deer in which prion seeding activity was detected in the tonsil and all three regions of the brain examined, yet all nasal secretion sampled tested were negative. Four deer that were positive in tonsil biopsies and the brain obex section (S8) had no positivity detected in either of the two rostral brain regions or in nasal swabs. We found no animals in which nasal swabs were positive and the brain sections were negative. In sham-inoculated control deer, all nasal swabs and tissues were negative by all assays.

Finally, consideration was given to the relationship of PRNP codon 96 polymorphism and clinical disease status for each deer. Thirteen of 18 deer were homozygous for glycine at codon 96GG. Eleven of the 13 exhibited clinical signs of disease (Table 1). In 10 of the 13 (77 %) deer, prion seeding activity was detected in nasal secretions. Two homozygous deer in which seeding activity was not detected using nasal swabs had clinical signs of disease and seeding activity in all brain sections. The remaining homozygous deer without detection in nasal secretions had no clinical signs of disease and was only positive in the obex brain section. Of the five 96GS deer, no seeding activity was detected in nasal secretions, even though two of the five had clinical signs of disease.

## Discussion

Given the continual spread and/or detection of CWD in three continents, a deeper understanding of prion excretion becomes more important, as does rapid methods to detect infection in live animals. Here we report that nasal swabbing can detect CWD prion shedding as early as 3 months after the first positive tonsil biopsy. However, we also found that detection can be intermittent and perhaps even absent in some animals. In this respect, it is pertinent to obtain serial (months apart) sampling of other secretions/excretions in CWD-infected deer with known point source exposure, in which serial tonsil and rectoanal mucosa-associated lymphoid tissue (RAMALT) biopsies were also monitored [19, 20]. It is of course difficult to distinguish whether negative results in animals known to be CWD-infected reflect limits in sensitivity, presence of inhibitors, both or just absence of prion shedding in that sample.

Previous work has estimated the concentration of prion shed in excreta to be log-fold lower than that in tissues [4, 11]. In the current study, longitudinal nasal sampling revealed intermittent positive results in five of ten deer, which could reflect varying levels of either shedding and/or assay inhibitors. Irregularities in detecting CWD prion shed in excreta have been demonstrated in multiple studies [21, 22], emphasizing the need for enhanced detection methods such as IOME RT-QuIC. Selective concentration of prion seeding activity in complex body fluids and excreta combined with amplification assays have aided non-invasive, relatively rapid detection in live animals [2–4, 22], yet challenges remain.

The efficacy of nasal swabs coupled with RT-QuIC analysis has been best demonstrated in the clinical detection of Creutzfeldt–Jakob disease (CJD), with sensitivity reaching 97 % [23–25]. Long nasal swabs assayed with RT-QuIC have also been shown to be positive in 60–83 % of deer and elk in clinical stages of CWD [6, 7]. In the present study, we found that nasal swabs taken near the end of study were positive in 10 of 18 deer (56%). Collectively, these minimally invasive approaches to detecting prion disease have found application in other protein misfolding diseases of humans, most notable thus far being Parkinson’s disease [26]. While this study was not designed to assess progression of CWD infection, the results suggest that CWD infection in the brain progresses in a caudal to rostral fashion and that involvement of the frontal cortex and olfactory bulb may be a precursor to shedding of prions in the nasal cavity.

The temporal correlations of prion seeding activity in nasal secretions with prion amplification in the brain suggests that nasal prions may be of central nervous system origin. Previous studies of transmissible mink encephalopathy (TME) and scrapie in hamsters and CWD in deer have strongly suggested a prion dissemination pathway from olfactory lobe neurons to the nasal olfactory mucosa [5, 27–32]. Our results reinforce these tenets. Given the long-demonstrated early replication of CWD prions in the tonsils, and the presence of nasal fluid positivity in some animals, it is still plausible that infected cervids shed prions of both neural and lymphoid origin.

Nasal swabs in the present work were collected from established cohorts of CWD-exposed deer in which infecting dose and stage of infection varied. Thereby, time of first shedding would be expected to vary, as reported in previous work [15]. The present results are consistent with the accumulating evidence that deer bearing the 96GS polymorphism experience a longer incubation time to first detection, a long course of CWD infection marked by lower levels of prion replication, and delayed and lower levels of prion shedding [33, 34]. We also observed increased nasal prion shedding as deer approached clinical stages of disease, consistent with the previous work by Haley *et al*. [6, 7].

Given the impact of seeding activity detection in other protein misfolding disorders of humans [23, 25, 26], continued studies on the detection and relationship of the nasal prions to progression of these disorders is merited.
